# Supplementary material for: The Molecular Mechanism of Positive Allosteric Modulation at the Dopamine D1 Receptor
Source: Int J Mol Sci. 2023 Aug 16;24(16):12848. doi: 10.3390/ijms241612848 (PMC10454769; doi:10.3390/ijms241612848)

Supplementary Information

Table S1. Standardized histidine states.

Histidine protonation states were standardized to those predicted by Protein Preparation Wizard using PROPKA pH 7.0.

Chain	Residue	Protonation State
G_α	H41	HID
	H64	HID
	H220	HID
	H357	HIP
	H362	HIP
	H387	HID
G_β	H54	HIE
	H62	HID
	H91	HID
	H142	HID
	H183	HID
	H225	HID
	H266	HID
	H311	HID
G_γ	H44	HID
D1R	H53	HID
	H164	HID
	H237	HID

Table S2. MM/GBSA calculations in the presence and absence of LY3154207.

There is no consistent trend in the sign of ΔΔG values, however both systems show a ΔΔG below 1.4 kcal/mol, which are smaller than the standard deviations in each condition.

OLBP	ΔG (SD) in the absence of LY315207	ΔG (SD) in the presence of LY315207	ΔΔG
Dopamine	-43.75 (3.38)	-45.11 (3.35)	1.36
Apomorphine	-64.70 (3.91)	-63.62 (4.58)	-1.08

Table S3. The network eigenvector centrality scores of the D1R/apomorphine systems.

Normalized eigenvector centrality scores for the D1R/apomorphine systems in the presence and absence of LY3154207. Only residues with an eigenvector centrality score of 0.10 or greater in any of the conditions are shown, and the score difference and absolute score difference are colored (green to yellow and gray to white) according to their respective maximum and minimum values.

Residue	BW index	D1R/ApoM	D1R/ApoM-LY	ApoM - ApoM-LY	abs diff
37	TM1.46	0.075	0.118	-0.043	0.043
41	TM1.50	0.109	0.130	-0.021	0.021
62	TM2.42	0.171	0.173	-0.002	0.002
63	TM2.43	0.110	0.112	-0.002	0.002
65	TM2.45	0.118	0.119	-0.001	0.001
66	TM2.46	0.213	0.207	0.006	0.006
67	TM2.47	0.087	0.095	-0.008	0.008
69	TM2.49	0.165	0.162	0.003	0.003
70	TM2.50	0.158	0.176	-0.018	0.018
72	TM2.52	0.096	0.099	-0.003	0.003
73	TM2.53	0.139	0.140	-0.001	0.001
77	TM2.57	0.090	0.093	-0.003	0.003
78	TM2.58	0.086	0.102	-0.016	0.016
105	TM3.34	0.091	0.100	-0.009	0.009
106	TM3.35	0.120	0.119	0.001	0.001
107	TM3.36	0.109	0.093	0.016	0.016
109	TM3.38	0.121	0.119	0.002	0.002
110	TM3.39	0.175	0.174	0.001	0.001
111	TM3.40	0.155	0.147	0.008	0.008
113	TM3.42	0.176	0.173	0.003	0.003
114	TM3.43	0.203	0.183	0.020	0.020
117	TM3.46	0.157	0.152	0.005	0.005
118	TM3.47	0.109	0.097	0.012	0.012
148	TM4.50	0.139	0.139	0.000	0.000
151	TM4.53	0.099	0.100	-0.001	0.001
203	TM5.47	0.114	0.107	0.007	0.007
206	TM5.50	0.105	0.100	0.005	0.005
210	TM5.54	0.143	0.129	0.014	0.014
214	TM5.58	0.101	0.082	0.019	0.019
281	TM6.44	0.154	0.140	0.014	0.014
285	TM6.48	0.127	0.122	0.005	0.005
321	TM7.43	0.111	0.115	-0.004	0.004
323	TM7.45	0.114	0.104	0.010	0.010
324	TM7.46	0.121	0.137	-0.016	0.016
327	TM7.49	0.154	0.152	0.002	0.002
328	TM7.50	0.091	0.106	-0.015	0.015
331	TM7.53	0.155	0.138	0.017	0.017

Figure S1. Stability of LY3154207 at IL2 in the D1R/apomorphine systems.

Cryo-EM structures of LY3154207 bound in the (A) horizontal orientation (PDB 7LJD). (B) Representative frames of the D1R/apomorphine-LY3154207 (horizontal) condition show that LY3154207 is stable in the horizontal orientation. (C) RMSDs for the horizontal PAM binding orientation plateau around 1.5 Å in all trajectories.

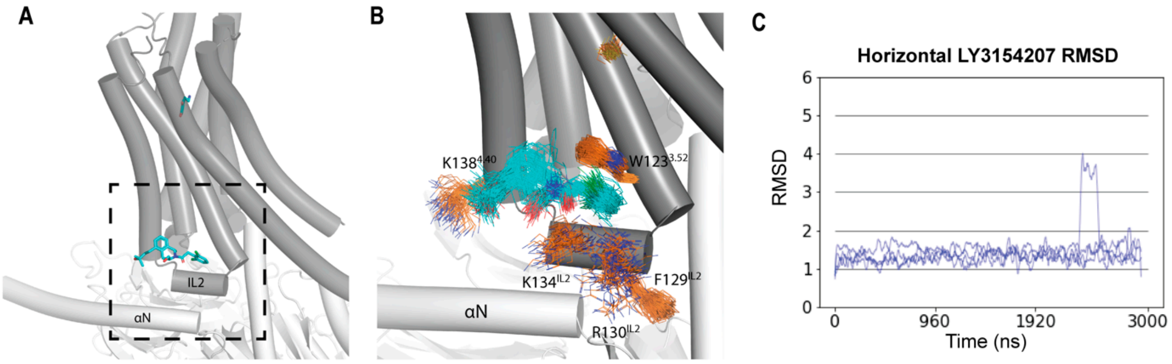

Figure S2. Stability of LY3154207 at IL2 in all simulated systems.

(A) The LY3154207 is divided into three substructures. RMSDs for the 3-hydroxy-3-methylbutyl tail (red), THIQ (blue), and dichlorophenyl ring (green) are plotted for both the D1R/dopamine-LY3154207 (B) and D1R/apomorphine-LY3154207 (C) conditions. (D) Pairwise RMSDs calculated as the average of ten 2000-frame random sample averages demonstrate considerable stability of the THIQ and dichlorophenyl ring moieties. Error bars represent the standard deviation across the 10 average pairwise RMSDs.

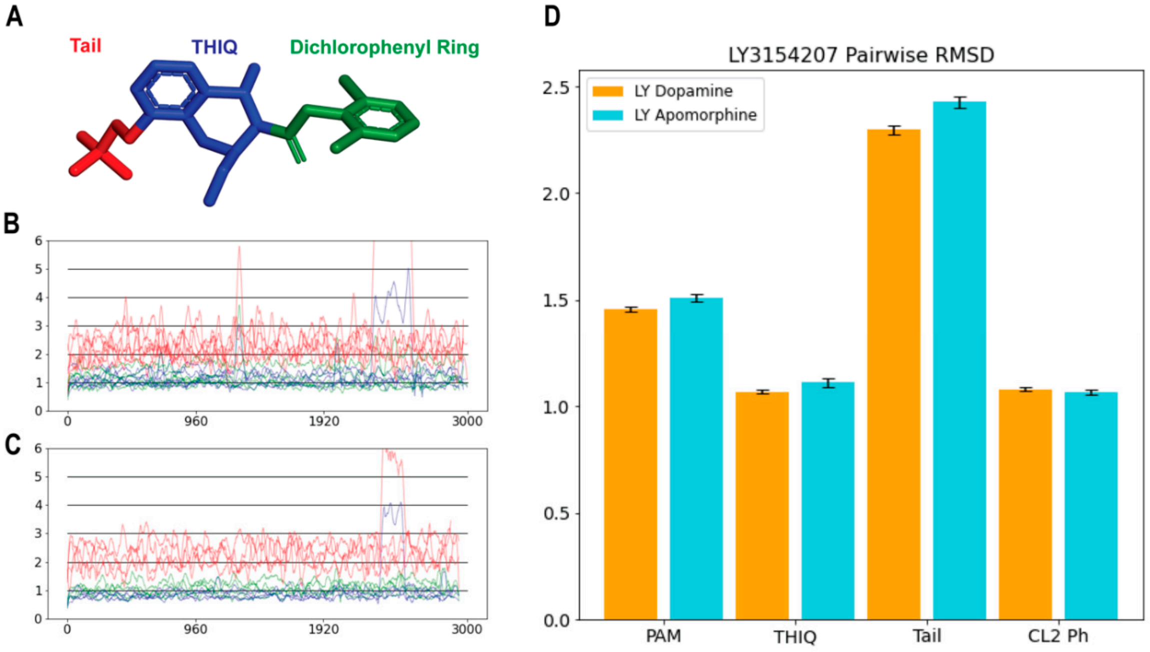

Figure S3. Recent cryo-EM structures of D1R/dopamine in the presence and absence of LY3154207 show nearly identical IL2 conformations.

A comparison between two recently published cryo-EM structures of the D1R/dopamine system aligned by the Cα atoms of the receptor residues in the presence (PDB 7X2F, shown in pale cyan) and absence (PDB 7F0T, shown in pale green) of LY3154207. Both structures are in the absence of GDP but in complex with Nb35.

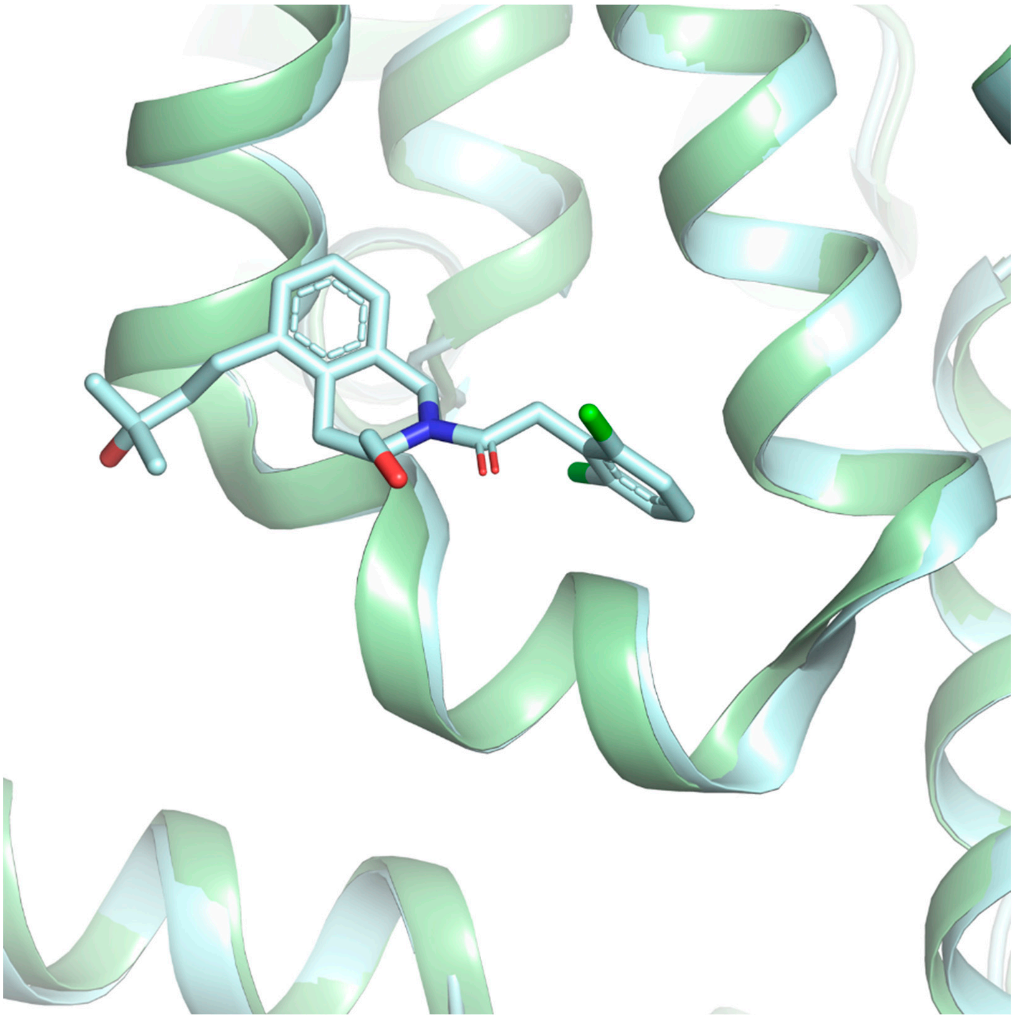

Figure S4. LY3154207 stabilizes a helical structure for intracellular loop 2 in the D1R/apomorphine system.

There is a greater helical frequency for residues P128, F129, and R130 when LY3154207 is bound (B) versus when it is not (A). In panels C and D, the evolutions of secondary structure of IL2 are shown for the entire representative trajectories of the D1R/apomorphine-alone and D1R/apomorphine-LY3154207 conditions, respectively. In the absence of PAM, this portion of IL2 could still adopt a helical conformation throughout the trajectory but less frequently. In panels A-D, helical and loop conformation assignments are indicated with red and gray, respectively. In panels E and F, residues $W123^{3.52}$, $R130^{IL2}$, $K134^{IL2}$, $K138^{4.40}$, and $A139^{4.41}$, which contribute to PAM binding, are shown in line representation for the D1R/apomorphine-alone (E) and D1R/apomorphine-LY3154207 (F) conditions. The portion of IL2 including residues 128-130 is highlighted in magenta.

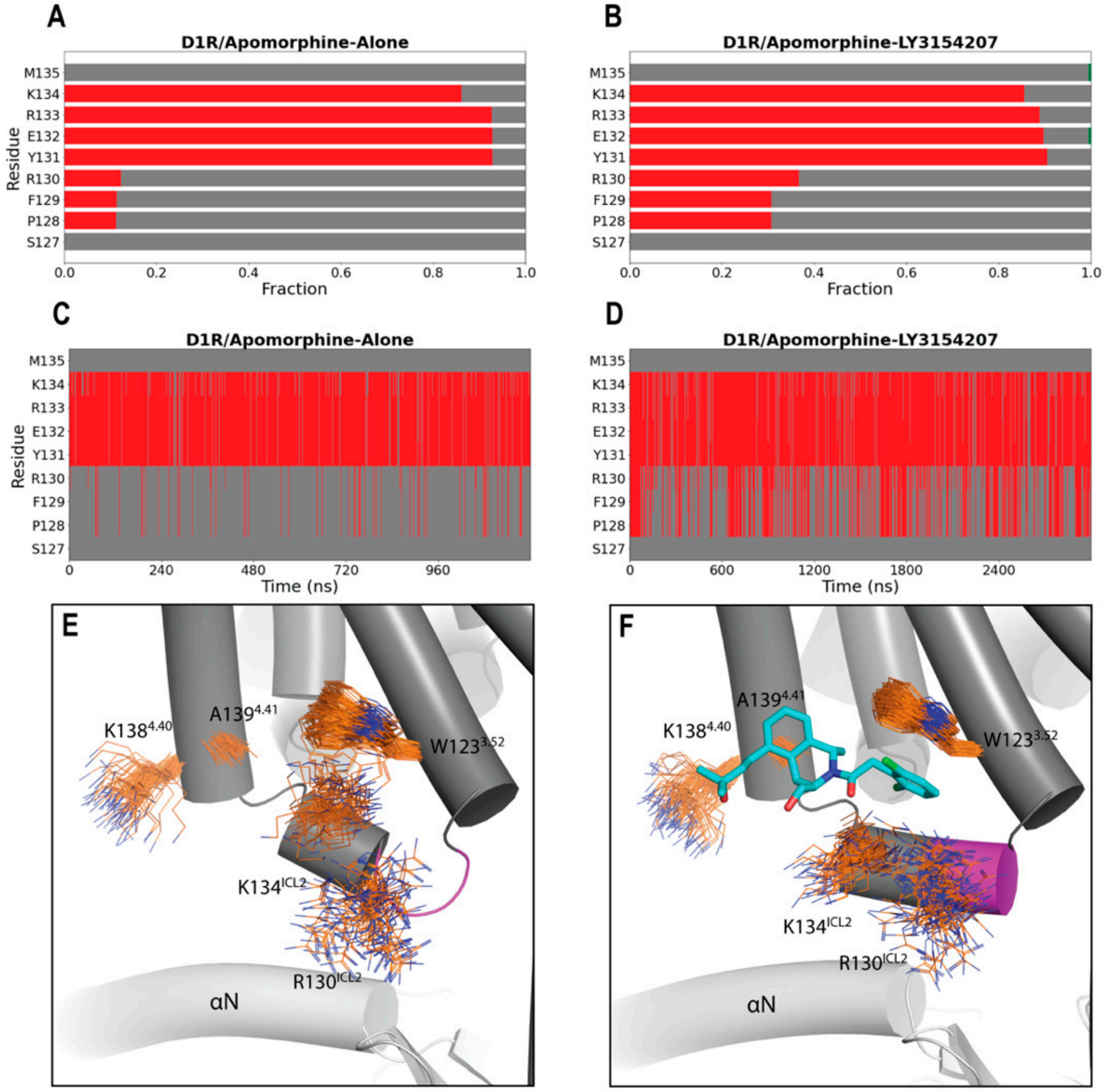
A
D1R/Apomorphine-Alone
M135
K134
R133
E132
Y131
R130
F129
P128
S127
Residue
0.0
0.2
0.4
0.6
0.8
1.0
Fraction
B
D1R/Apomorphine-LY3154207
C
D
Time (ns)
0
240
480
720
960
600
1200
1800
2400
E
F
K138$^{4.40}$
A139$^{4.41}$
W123$^{3.52}$
K134^{ICL2}
R130^{ICL2}
αN

Figure S5. Pairwise RMSD for ligands bound in the OLBP in the presence and absence of LY3154207.

The ligand in the OLBP is stable with pairwise RMSDs below 1.2 Å. PAM binding has no significant effect on the stability of dopamine in the OLBP, however there is an increase in the pairwise RMSD for apomorphine in the D1R/apomorphine-LY3154207 condition. Each bar represents the average of average pairwise RMSDs from ten 2000-frame random samples, and error bars represent the standard deviation across the ten averages.

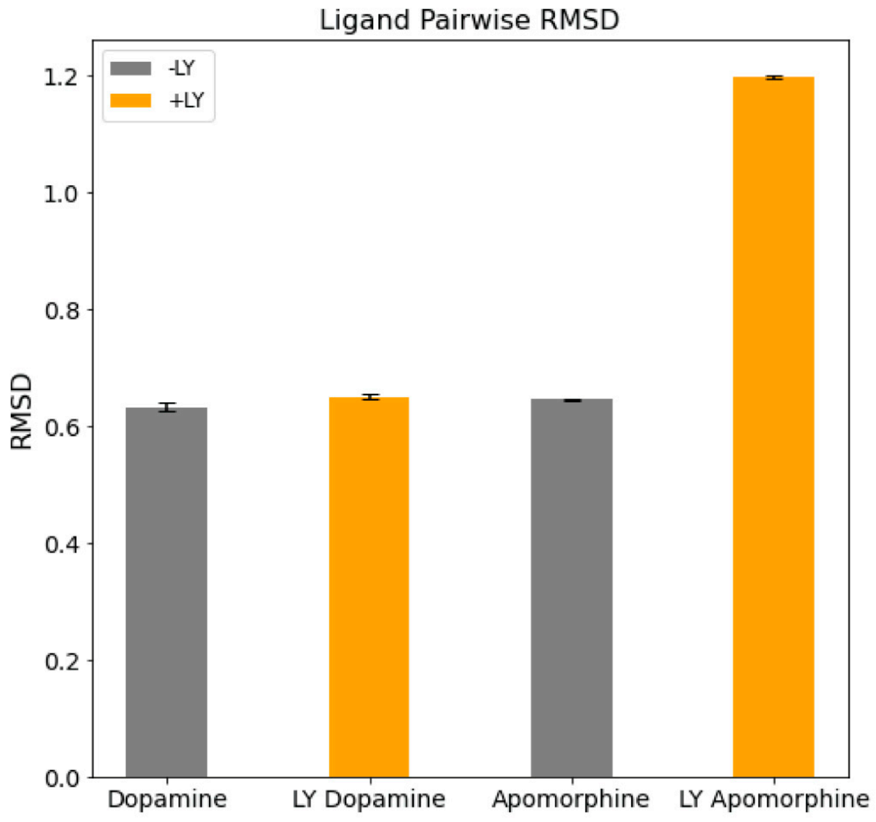

Figure S6. Network analysis identified impacts on several key structural motifs in the presence of LY3154207 in the D1R/apomorphine system.

(A) A network connecting residues with larger than 0.2 contact frequency in the D1R/apomorphine-LY3154207 condition. If the shortest heavy-atom distance between two residues was within 4.5 Å, we defined that they formed a contact. Edge radii are scaled by contact frequency. (B) Top and (C) side views of the LY3154207-bound D1R/apomorphine system with nodes colored and scaled in radius according to the eigenvector centrality score. All nodes with an absolute score difference exceeding 0.005 are highlighted in color. Eigenvector centrality analysis demonstrates that in the presence of LY3154207, the D1R/apomorphine system has higher scores (yellow) for residues in the extracellular region enclosed by TMs 1, 2, 3, and 7, and lower scores (green) for residues in the intracellular region.

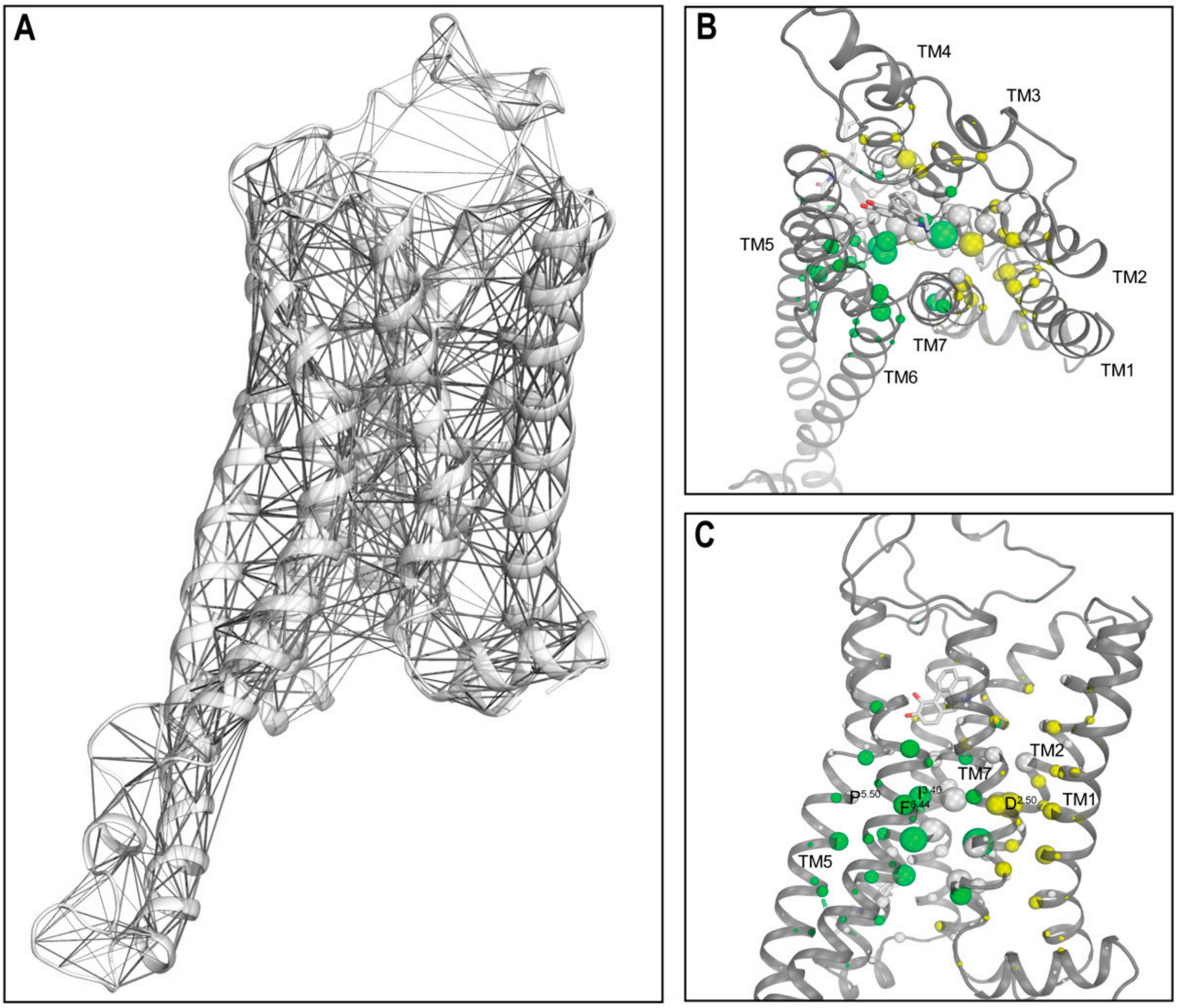
A
B
TM4
TM3
TM5
TM2
TM7
TM1
TM6
C
TM2
TM7
TM1
TM5

Figure S7. LY3154207 binding is associated with a tightening and loosening of the extracellular and intracellular regions of the receptor, respectively.

(A) An overview of the D1R coupled with Gs protein and in complex with both apomorphine and LY3154207. The extracellular and intracellular subsegments of the D1R are colored in white, while the middle segments are in gray (see Methods for their definitions). Our PIA analysis demonstrates that the majority of extracellular subsegments have a tendency to move closer together (B), while the majority of intracellular subsegments have greater COM-COM distances (C) in the D1R/apomorphine-LY3154207 compared to the D1R/apomorphine-alone condition.

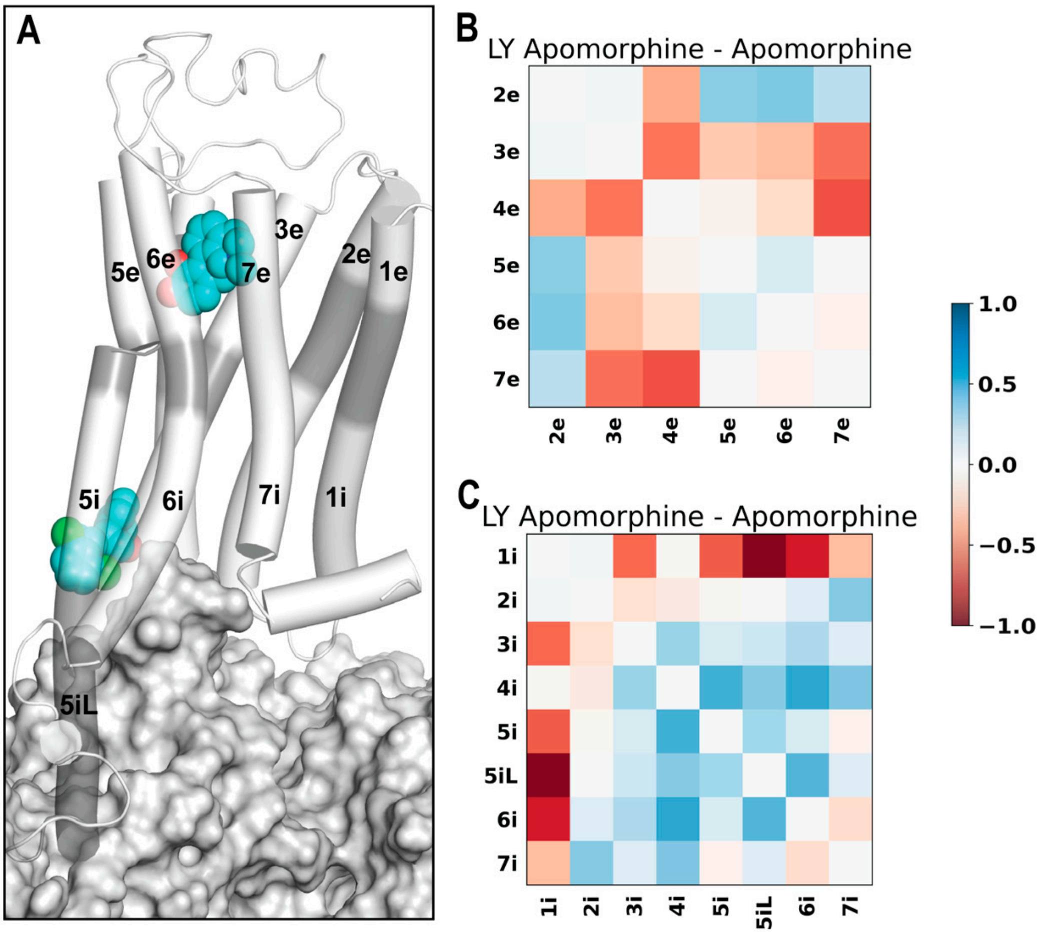

Figure S8. Contact frequency of receptor and $G_{s\alpha}$ residues in the D1R/apomorphine system.

(A) The contact map for the interface between D1R/IL2 and Gα residues. Cells containing an 'X' represent the corresponding residue pairs forming a contact in both the D1R/apomorphine-LY3154207 and D1R/apomorphine-alone conditions. More frequent interactions in the presence of PAM are colored in yellow, and less frequent interactions are in green. Interactions are mapped on the D1R/Gα model shown in panel B. (B) In the presence of PAM, IL2 makes more consistent interactions with αN of Gα, specifically $R133^{IL2}$. (C) The contact map for the interface between D1R/TM5iL and Gα residues is mapped to the model in panel (D). All contacts with a frequency difference greater than or equal to 0.10 are shown, and tubes are scaled to the frequency difference.

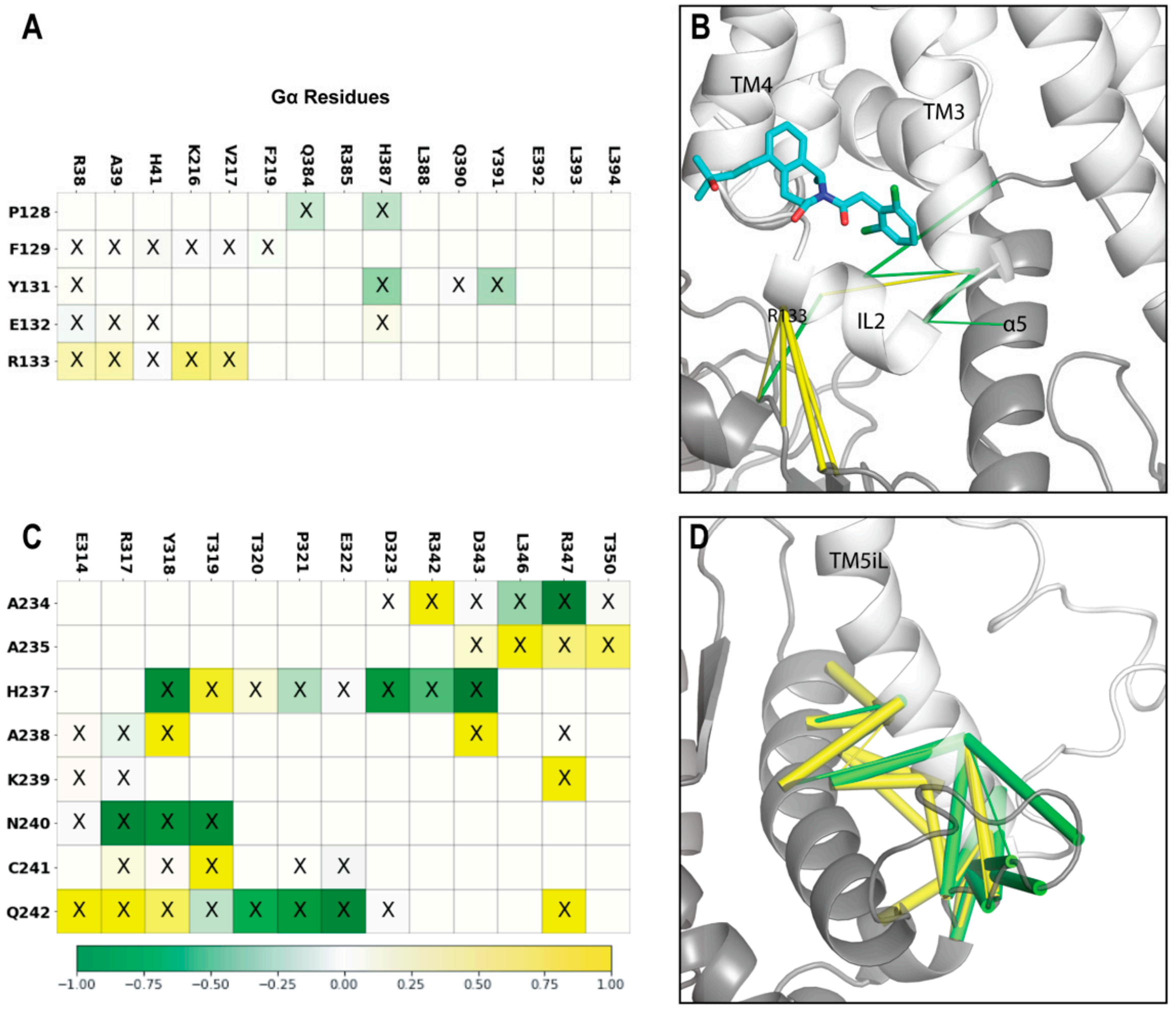
A
Gα Residues
R38 A39 H41 K216 V217 F219 Q384 R385 H387 L388 Q390 Y391 E392 L393 L394
P128
F129
Y131
E132
R133
B
TM4
TM3
R133
IL2
α5
C
E314 R317 Y318 T319 T320 P321 E322 D323 R342 D343 L346 R347 T350
A234
A235
H237
A238
K239
N240
C241
Q242
−1.00 −0.75 −0.50 −0.25 0.00 0.25 0.50 0.75 1.00
D
TM5iL

Figure S9. LY3154207 binding induces a conformational change in IL3.

Secondary structure assignment for residues 236-268 of IL3. Helix, loop, and sheet assignment are depicted as red, gray, and green, respectively. In the D1R/dopamine (A) and D1R/apomorphine (B) systems, there is no convergent IL3 conformation. In the presence of LY3154207, the D1R/dopamine (C) and D1R/apomorphine (D) systems have similar IL3 conformations. Specifically, short helical segments emerge in IL3 for residues 245-248 and 250-254. Red bars are shown to the side of (C) and (D) to highlight regions that more frequently adopt a helical structure in the presence of LY3154207.

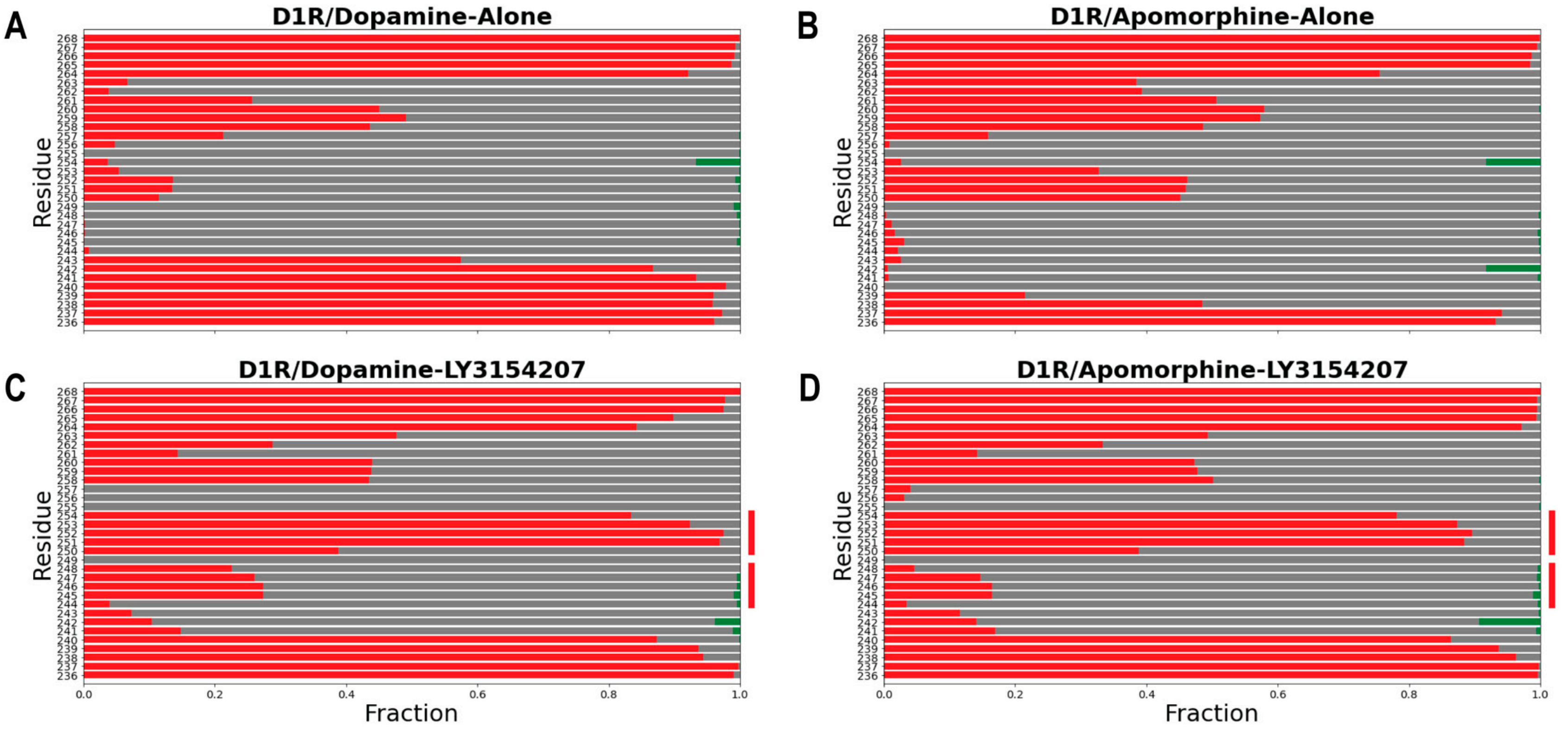

Supplement: Supplementary file 1 [file ijms-24-12848-s001.zip › ijms-2558636-supplementary.pdf]
